# Flexible potentiometric pH sensors for wearable systems

**DOI:** 10.1039/d0ra00016g

**Published:** 2020-02-27

**Authors:** Libu Manjakkal, Saoirse Dervin, Ravinder Dahiya

**Affiliations:** Bendable Electronics and Sensing Technologies (BEST) Group, School of Engineering, University of Glasgow G12 8QQ UK Ravinder.Dahiya@glasgow.ac.uk

## Abstract

There is a growing demand for developing wearable sensors that can non-invasively detect the signs of chronic diseases early on to possibly enable self-health management. Among these the flexible and stretchable electrochemical pH sensors are particularly important as the pH levels influence most chemical and biological reactions in materials, life and environmental sciences. In this review, we discuss the most recent developments in wearable electrochemical potentiometric pH sensors, covering the key topics such as (i) suitability of potentiometric pH sensors in wearable systems; (ii) designs of flexible potentiometric pH sensors, which may vary with target applications; (iii) materials for various components of the sensor such as substrates, reference and sensitive electrode; (iv) applications of flexible potentiometric pH sensors, and (v) the challenges relating to flexible potentiometric pH sensors.

## Abstract 2

This review presents recent progress, importance, requirements and future needs of wearable potentiometric pH sensors for healthcare applications.

## Graphical abstract

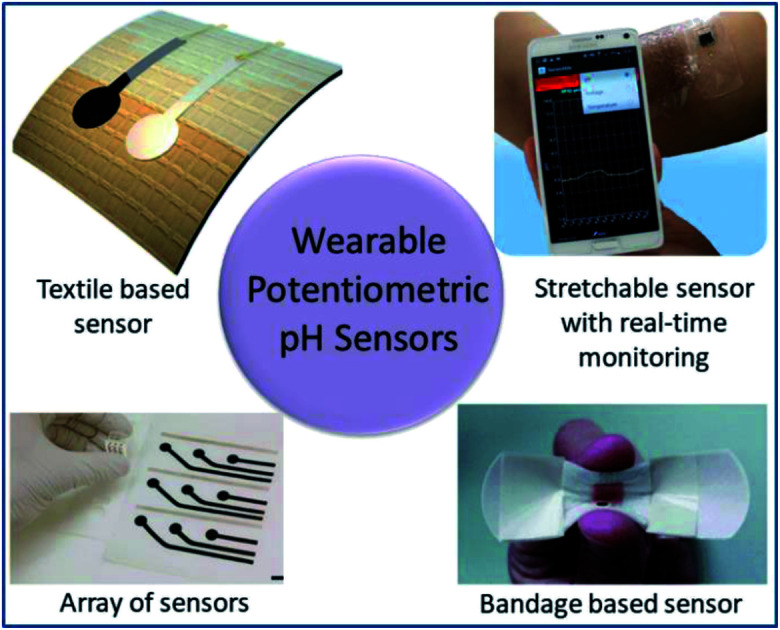

## 1. Introduction

Chronic diseases, including diabetes, cancer, cardiovascular disease and mental health disorders are the leading cause of death and disability worldwide.^1–3^ The early and real-time detection of the physicochemical and biological representations of these diseases could ensure rapid and efficient patient treatment, thus leading to positive health impacts. Despite recent technological advances, traditional methods of laboratory-based disease diagnosis are impeded by their long turn-around time. Current approaches for the detection, identification and treatment of chronic diseases require a biofluid sample to be obtained from the patient, before being sent to the lab for analysis, after which the result is forwarded to a physician for consideration and patient communication. This process can range from a number of hours to several days depending on the characteristics of the target analyte, the measurement performed, and the level of expertise required to assess and disseminate results. Easy to use and rapid diagnostic technologies are therefore required to ensure fast-moving disease diagnosis and treatment. In this regard, wearable devices integrated with advanced sensing technologies that are capable of real-time physicochemical and biological measurements have gained interest.^4–6^ Wearable sensors that can be attached to the body or even integrated into clothing have opened up interesting opportunities in terms of non-invasive health monitoring.^7–13^ For example, the acute physiological responses obtained with such sensor systems have significantly improved our understanding of diseases, including heart failure, epilepsy and Parkinson's disease.^14–16^ Considering that potential, numerous health-monitoring devices in the form of wearable pads, wristbands and straps *etc.* have been developed for the measurement of several physiological functions and parameters including heart rate, rhythm and electrical activity, respiration rate, blood pressure and skin temperature, *etc.*^17–20^

The development of electrochemical sensors on flexible and compliant substrates has added a new dimension to wearable healthcare technologies by facilitating conformability to the body and reduced interference from non-target analytes or contaminants.^8,10,21–26^ These sensors can measure a wide range of physicochemical and biological body parameters such as pH, glucose, urea, salinity, Ca^2+^ and dopamine levels, *etc.*, which are important for disease monitoring and diagnosis.^8,10,23,27–31^ Among various flexible and stretchable electrochemical sensors, pH sensors are particularly important as pH levels influence most chemical and biological reactions in materials, life and environmental sciences. Conventional glass pH electrodes are not suitable for wearable systems due to their lack of bending capability and the fact that glass can easily crack during user movement.^32^ In addition, glass pH sensitive electrodes are large in size, are difficult to be miniaturized, and require regular topping up of the reference buffer solution.^33–35^ As a result, alternative technologies such as solid-state pH sensors are being explored.^8^ Besides the possibility of miniaturization and flexibility, they offer several attractive features such as faster response, wider pH sensing range, excellent sensitivity, simple electronics, biocompatibility, low cost of fabrication, and the possibility of integration on different substrates (polymer, plastic, textiles, paper, *etc.*). For these reasons the development of solid-state pH sensors on various flexible substrates using sensitive materials ranging from metal oxides (MO_*x*_) and semiconductors to polymers and carbon-based materials is a growing area of research in electrochemical sensing technologies.^8^ Electrochemical pH sensors of this kind can generally be categorized as potentiometric,^36,37^ conductimetric/chemi-resistors^38–41^ or ion sensitive field effect transistors (ISFET)^42–44^*etc.* The potentiometric pH sensors are discussed here in detail (Section 3 given design) as they offer stable performance, high sensitivity, less interference, easiness in wireless system, low power, long lifetime and fast response as compared to other type of pH sensors.

This review article presents the recent progress, importance, requirements and future needs of wearable potentiometric pH sensors, in addition to the application of such with particular reference to healthcare applications. A summary of the design and components of state-of-the-art flexible potentiometric pH sensors are presented along with the gaps or challenges to be addressed. Unlike other reviews articles on polymer-based pH sensors,^45^ microfabricated electrochemical pH sensors^46^ and MO_*x*_ based sensors,^32^ this article focuses on flexible potentiometric pH sensors for wearable, their design and components, applications, challenges and the future outlook. A substantial part of the discussion focuses on the design of flexible potentiometric pH sensors, with emphasis on flexible substrates, the materials for the reference and sensitive electrodes, sensitivity and biocompatibility – which is paramount to the success of wearable pH sensors for health applications.

## 2. Importance of wearable pH sensing for health monitoring

Body fluids such as sweat, tears, saliva, and urine *etc* contain a wide range of health related biomarkers (*e.g.* small molecules (cortisol, glucose, urea, lactate, uric acid), ions (H^+^, Na^+^, K^+^, Ca^2+^, NH^4+^, Cl^−^) and peptides or small proteins (neuropeptides and cytokines) *etc.*).^47^ Furthermore, the physiochemical and biological composition of these body-fluids contains a treasure trove of suggestive analytes, including hydrogen ions, that can be used to establish the status of an individual's health and/or several health ailments.^8^ Such approaches could promote non-invasive health monitoring and potentially eliminate the need for acquiring blood samples for analysis. As a result, wearable health monitoring systems using body fluids have recently attracted significant interest. Among various physiochemical and biological health markers, pH measurements can reveal a wealth of information about the physiological status of an individual. For example, pH levels can reflect variations in the local, regional, and systemic acid–base balance which is related to health and disease.^48^ Furthermore, the variation in pH values can reflect the activity of many physiological, biological and medical processes such as enzymatic reaction, tumour metastasis,^49,50^ wound healing^51,52^ cellular growth^13,53^*etc.* Therefore, *in situ* assessment of the pH levels of body fluids, such as sweat, tears, urine and saliva could provide the relevant information necessary for the early detection of many diseases.^54^ In particular, real time sweat analysis has attracted interest for the detection and monitoring of many relevant analytes, including pH, Na^+^ and Cl^−^ ions, alcohol and glucose levels.

The pH of sweat can provide information about the physiological status of disease or infection (Table 1).^47^ For example, patients with cystic fibrosis have alkaline sweat (up to pH 9) as compared to healthy persons (pH range 4.5–6.5), due to the defect in bicarbonate reabsorption. Likewise, in the case of an infected wound, the pH value is in the range of 7–8.5 due to the presence of bacterial colonies and enzymes. The pH of sweat is also directly related to the pH of our skin and so, variations in sweat pH can reflect pathogenesis and skin diseases such as irritant contact dermatitis, atopic dermatitis, acne vulgaris, *etc.*^77^ Some studies show that sweat is also closely correlated to blood glucose levels.^78^ Furthermore, sweat pH levels reflect the intensity of physical exercise an individual has undergone and the level of dehydration *etc.* For example, during physical exercise, the concentration of NH_3_ in sweat decreases as it is transformed into ammonium (NH_4_^+^) ion. Since NH_4_^+^ is less diffusible across cellular membranes as compared to NH_3_, the excess NH_4_^+^ lead to an increase in sweat pH.^8,79,80^

**Table 1:** The importance of physiological pH monitoring

Body fluid	Function	Balanced pH	pH imbalance	Physiological Status	Ref.
Saliva	• Maintain healthy mouth	6.2–7.6	Acidic (<pH 5.5)	• Demineralization and the breakdown of tooth enamel	55–60
				• Mineral deficiency (*e.g.* calcium and magnesium), often due to poor digestion	
				• Chronic generalized periodontitis	
	• Protect teeth		Alkaline (>pH 5.5)	• Plaque formation	
				• Chronic generalized gingivitis	
Tears	• Prevent eye dryness	6.5–7.6	Acidic (<pH 5.5)	• Chemical damage	61–64
			Alkaline (>pH 5.5)		
Urine	• Excrete waste fluid from the kidneys	4.5–8.0	Acidic (<pH 5.5)	• Metabolic syndrome	65–69
				• Diabetic ketoacidosis (a complication of diabetes)	
				• Idiopathic uric acid nephrolithiasis (the process of forming a kidney stones)	
				• Diarrhea	
				• Starvation	
			Alkaline (>pH 5.5)	• Kidney stones	
				• Kidney-related disorders	
				• Urinary tract infections (UTIs)	
Sweat	• Control body temperature	4.5–7.0	Acidic (<pH 5.5)	• Acidosis	70–76
				• Excessive sweating	
			Alkaline (>pH 5.5)	• Electrolyte imbalance	
				• Cystic fibrosis	
				• Physical stress	
				• Osteoporosis	
				• Bone mineral loss	

**Fig. 1:**
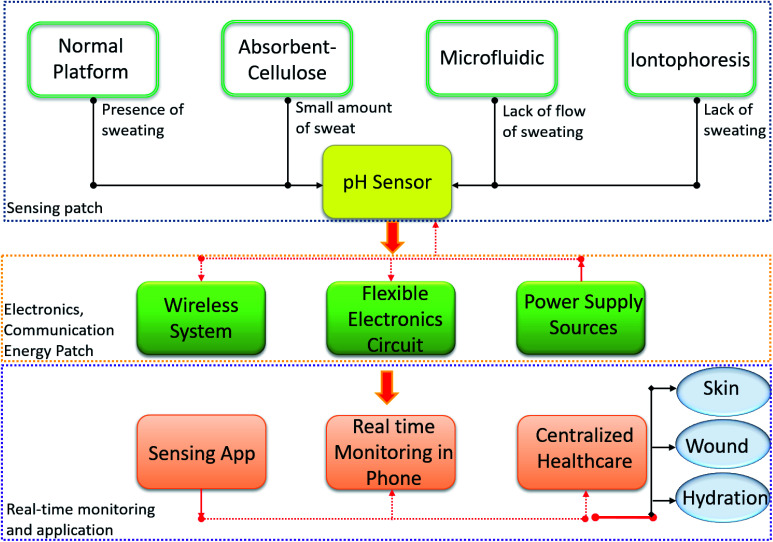
Wearable pH sensor requirement for real time sweat monitoring.

Similarly, other biofluids such as ocular fluid, urine and saliva can also provide clinically relevant information for medical diagnosis.^54,81,82^ As complex extracellular fluids, tears contain quantities of blood and can be an attractive fluid for non-invasive health care monitoring.^61^ Tears also contain antioxidants, amino acids, proteins, peptides, electrolytes, lipids, metabolites and various ocular cells.^62–64^ Sensors based on soft contact lenses with integrated wireless electronics have recently been explored to monitor analytes found in tears.^83^ Tears can reveal information regarding systemic disorders, including diabetes and ocular conditions (Table 1).^84–86^ For instance, a decrease in tear pH may suggest a corneal infection before symptoms are visible.^87^ However, among the many ionic species found in tears, pH is least often measured due to the low concentrations of H_3_O^+^ present. It is also difficult to collect sufficient tear fluid for efficient pH measurements.^87,88^ Similar challenges exist with sweat as it is not always available in sufficient amounts (discussed in detail in Section 4 of this review). To realize an efficient and compliant wearable real time pH monitoring platform, it is important to solve the current issues facing these innovative technologies, a summary of such issues is described for the case of sweat pH analysis in Fig. 1.

The implementation of wearable potentiometric sensors for urine assessment has also previously been demonstrated. Urinary pH analysis can be used to monitor kidney functionality, renal tubular acidosis, hydration, and other urinary tract-related issues (Table 1).^89^ In fact, pH is a routine clinical investigation performed to assess kidney functionality.^90^

In comparison to blood, sweat and urine, saliva is a more readily available body fluid, especially for patients suffering from sweat inhibiting conditions or those who suffer from chronic renal failure.^56,91^ The real-time chemical analysis of saliva could offer an excellent non-invasive method for monitoring the emotional, hormonal, nutritional, and metabolic state of the body.^55^ The ready availability of saliva is a clear advantage for mobile healthcare and home medical devices. For example, real-time monitoring of saliva pH levels could facilitate the self-management of oral conditions like dental and enamel erosion^92,93^ Furthermore, pH measurements of saliva can be used as a clinical indicator of certain diseases including diabetes, inflammation and infection and have thus been increasingly used by researchers and clinicians as tools for routine dental and medical examinations.^91,94,95^ For instance, gastroesophageal reflux patients exhibit a low saliva pH.^96–99^ Low saliva pH is also associated with the presence of mucosal stomatitis.^91,100^ Thus, reliable measure of the pH of various body fluids could provide useful information about health monitoring and this can be achieved using potentiometric pH sensors. Depending on the applications in wearables, the standard design of the shape or array of electrodes in potentiometric sensors could vary. Substrates play crucial role for such designs and this is discussed in the following section.

## 3. Substrates for potentiometric pH sensor

### 3.1. Substrates for flexible sensors

Although the structural components of pH sensors, including the substrate, dielectrics, passivation and encapsulation layers are not directly involved in pH sensing, they offer structural support, protection to non-sensing elements and couple device electrical signals and are thus indispensable to device functionality.^45^ The substrate, which is often a flat, solid-state platform that facilitates the processing of sensitive materials, significantly influences the sensors' physical, mechanical, and electrical features.^45,101^ Various substrates used for pH sensors fabrication are discussed in this section.

#### 3.1.1. Polymer based substrates

**Fig. 2:**
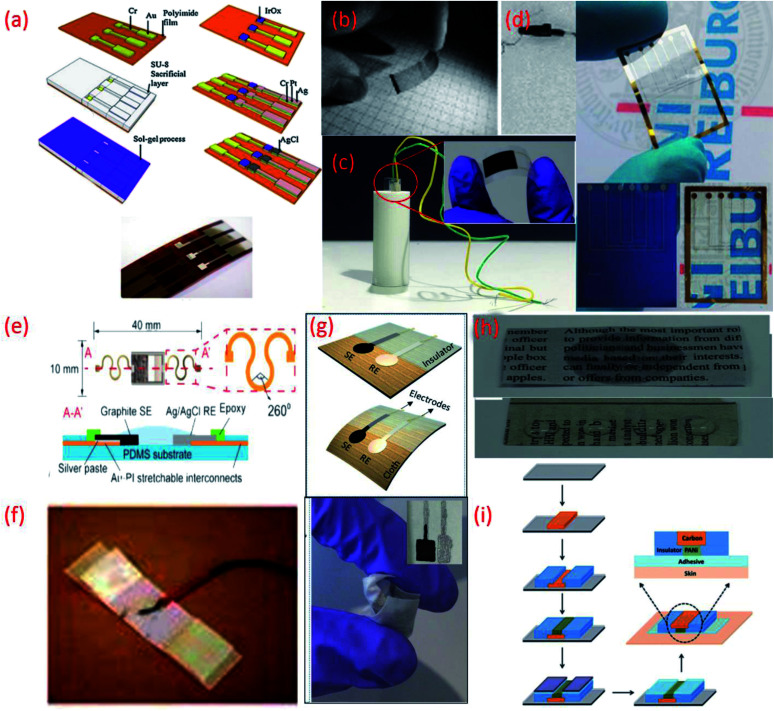
(a) Fabrication processes of IrO_2_ based pH sensor on a polyimide substrate, Reprinted from Publication^115^ with permission from Elsevier. (b) Transparent and flexible CNT/PANI pH sensors made on PET and PVC coated steel wire substrates.^125^ (c) Image of flexible sensor made on PET substrate (inset) fitted in a tube with a radius of curvature 5 mm.^22^ (d) Flexible ZnO TF-FET pH sensor on Si/SiO_2_ and on PEN (transparent) substrates. (e) Stretchable pH sensors on PDMS substrate Reprinted from Publication^8^ with permission from Elsevier. (f) Sensor on a yarn substrate attached on band-aid.^132^ (g) Graphite and Ag/AgCl based pH sensor on cellulose/polyester cloth substrate.^133^ (h) Photographs of pristine newspaper and PC-paper for pH sensor fabrication Reprinted from Publication^134^ with permission from Elsevier. (i) Schematic representation of the fabrication steps of the sensors on tattoo Reprinted with permission from Publication.^135^

Due to their chemical inertness and thermal and electrical insulative capacities, polymeric materials such as polyimide (PI), polyethylene terephthalate (PET) and polyethylene naphthalene (PEN) have been used as substrates for wearable electronic devices. In fact, due to these features they are widely used in flexible electronics.^102–110^ The user comfort and safety are also important prerequisites for on-body sensors and substrates. In this regards, biocompatible polymeric substrates such as polydimethylsiloxane (PDMS) have been explored for the fabrication of pH sensors.^101,103,111,112^ Among the many available polymeric substrates, PI, which is commercially available as Apical, Kapton, and UPILEX films, *etc.*, presents impressive bendability, good mechanical strength, dimensional stability, low surface roughness, excellent electrical properties and low dielectric constant (Table 2).^45,102,113^ It also displays good thermal and chemical stability resisting weak acids, alkalis and commonly used organic solvents.^102^ Furthermore, PI substrates are durable when deformed, thus allowing the sensors to retain the pH sensitivity under bending conditions.^114^ Early use of PI as a flexible substrate for potentiometric pH sensors established a simple yet flexible sol–gel processed iridium oxide (IrO_*x*_) based pH sensor (Fig. 2a).^115^ The sensors demonstrated distinct response potentials at various pH levels when conformed to the curved inner surface of a glass test tube. Due to their attractive flexibility, PI supported IrO_*x*_ sensing films have also been employed to monitor pH levels within a live pig's oesophagus and a rabbit and human heart.^116–118^ Another example of the use of Kapton as a flexible substrate is the development of a wearable multiparametric smartwatch with an array of 16 epidermal sensors that can be mounted directly onto the user's wrist to monitor pH levels in sweat.^119,120^ It should be noted however that PI is a relatively expensive substrate. Furthermore, the poor adhesion strength of PI often results in poor interaction with other deposited materials^45,102,121,122^ and its yellow-brown colour yields low device transparency.^102^

**Table 2:** Properties of the substrates and platforms used for wearable/flexible electrochemical sensor fabrication

Substrate	Properties	Ref.
Polyimide (PI) (Apical, Kapton, UPILEX films, *etc.*)	• Bendable	124, 138–141
	• Low transparency	
	• Dielectric constant 2.8–3.5	
	• Resistant to temperature <450 °C	
	• Coefficient of thermal expansion ≈5 × 10^−5^ K^−1^	
	• Resistant to weak acids and alkalis	
	• Resistant to ethanol and acetone	
Polyethylene terephthalate (PET)	• Bendable	123, 142 and 143
	• >85% transparency	
	• Dielectric constant 2.5–3.5	
	• Resistant to temperature <100 °C	
	• Coefficient of thermal expansion ≈7 × 10^−5^ K^−1^	
	• Dissolvable in acetone	
Polyethylene naphthalene (PEN)	• Bendable	124 and 144
	• >85% transparency	
	• Dielectric constant 2.9–3.2	
	• Resistant to temperature <180 °C	
	• Coefficient of thermal expansion ≈2 × 10^−5^ K^−1^	
	• Easily permeated by oxygen and water	
Polydimethylsiloxane (PDMS)	• Stretchable	145–148
	• >95% transparency	
	• Dielectric constant 2.3–2.8	
	• Resistant to temperature <100 °C	
	• Coefficient of thermal expansion ≈30 × 10^−5^ K^−1^	
	• Mostly resistant to ethanol and acetone, may cause swelling	
Fibers, textiles & fabrics	• Stretchable & bendable	132, 133 and 149
	• Low transparency/opaque	
	• Resistant to temperature <100 °C	
	• Easily permeated by oxygen and water	
Tattoos	• Bendable	135 and 150
	• Stretchable	
	• Opaque	
	• Resistant to temperature <100 °C	
Paper	• Bendable	103 and 151
	• Opaque	
	• Resistant to temperature <100 °C	
	• Dielectric constant 2.3–3.0	
	• Absorbs moisture	
	• Dissolves in strong acids	
Bandage	• Stretchable & bendable	10 and 152
	• Opaque	
	• Resistant to temperature <100 °C	
	• Easily permeated by oxygen	
	• Absorb moisture	
	• Water soluble/waterproof	

On the other hand polyester PET films and PEN, an insulating, transparent and mechanically flexible plastic foil are both relatively low-cost substrates that transmit >85% of light in the visible wavelength region.^102,123,124^ However, due to their relatively high modulus of elasticity both PET and PEN present relatively poor stretchability. Nonetheless, several interesting pH sensors have been developed using these substrates. For example, PET and a PVC coated steel wire were used to deposit a CNT network coating for optical and potentiometric pH measurements (Fig. 2b).^125^ Using these types of substrates helps overcome problems relating to sensor size and stiffness and opens a wide range of applications for electrochemical sensors. More recently, PET has been exploited for the development of a low-cost pH sensor fabricated using a simple screen-printing process and dilute chemical polymerization.^126^ PET has also been demonstrated as a suitable substrate to support the printing of MO_*x*_ (CuO) based sensitive electrodes. The flexibility of the PET supported sensor was studied by inserting the sensor into a tube of 5 mm (Fig. 2c) and 10 mm radius of curvature, respectively, to demonstrate the impedance and capacitance variations of the sensor due to bending.^22^ Wearable glucose sensors that provide highly sensitive pH monitoring in the range of pH 5–9 have also been developed using both PET and poly (diallyldimethylammonium chloride) (PDDA), a cationic, water soluble polyelectrolyte as substrates.^127^ PEN substrates have also been used to develop small volume, potentiometric zinc oxide (ZnO) thin film-FET (TF-FET) pH sensors^128–130^ (Fig. 2d). PDMS substrates have also been widely adopted for the development of flexible and stretchable wearable devices due to high intrinsic stretchability and biocompatibility.^102^ Due to these features, PDMS has been regarded as an ideal candidate for the development of wearable and disposable electrochemical glucose and pH sensing devices.^131^ The flexible design of this particular point-of-care therapy device facilitates conformal skin contact, which facilitates a high performance under physical deformation and promotes sufficient sweat collection. PDMS was also employed as a substrate for the development of a stretchable system for wireless monitoring of pH from sweat (Fig. 2e).^8^

#### 3.1.2. Fabric based substrates

Commodity materials such as fibres, yarns, textiles and cloth based fabrics provide an affordable platform for the development of state-of-the-art wearable devices with enhanced sensing functionalities.^136^ A variety of fibres fashioned from natural materials, including wool, cotton and silk, as well as from synthetic materials, such as nylon, polyester, spandex and carbonaceous materials are available as multi-functional wearable device substrates.^136^ Having similar elastic properties as the human skin, many of these highly desirable, large area interfaces can improve conformal contact between the sensing device and the users skin.^137^ Their breathable and conformal nature also boosts transpiration of the body fluids often used to generate health-related physiological signals.^137^ The intrinsic biocompatibility of the materials used to shape fibres, textiles and fabrics also eliminates the potential safety risks associated with many conventional substrates. In addition, by using clothing as a substrate, sensors can be seamlessly integrated into apparel as discreet form factors for functional fashion.^136^ In recent times, multifunctional fabric-based devices, composed of conductive yarns and even smaller fibres have been developed for a number of applications, including health-care monitoring, by surface-mounting fully formed sensors or functional materials onto commercially available fabrics in a layer-by-layer fashion.^136,153,154^ So far, many studies have devoted their focus to the modification of cotton yarns. One flexible, ion-selective pH sensor employed for pH, K^+^ and NH_4_^+^ was constructed by using a CNT ink to turn cotton yarns into ion-to-electron transducers.^132^ The overall simplicity of this fabrication approach and operation of the sensor is attractive for the potential mass production of disposable, wearable devices (Fig. 2f).^132^

Another reported wearable potentiometric pH sensor on textile based substrates uses a graphite-polyurethane (G-PU) thick-film SE on a cellulose–polyester blend cloth (Fig. 2g).^133^ The mechanical stability and strong adhesion of the SE is attributed to use of the polyurethane (PU) binder which, unlike other binders, offers excellent structural support and flexibility. Hydrogen bonds established during the polymerization of urethane end groups also enhance contact between PU and the cellulose–polyester blend cloth.^155,156^ Such excellent adhesion allows the textile-based sensor to be washed without affecting the pH response.

#### 3.1.3. Paper based substrates

Paper-based materials have attracted a great deal of interest for their role as substrates in wearable sensing devices because they are inexpensive, readily available, bendable, light weight, biocompatible, biodegradable and sustainable.^45,157–159^ With their unique morphologies and various pore sizes, paper based substrates can also facilitate the transportation of fluids, using passive pumping, a phenomenon that uses pressure differences induced by surface tension to drive the movement of fluid in closed channels.^159,160^ Additionally, hydrophilic or hydrophobic patterning *via* photolithography can be used to fabricate paper-based devices with microfluidic channels.^45,159^ Due to their degradability, paper-based potentiometric pH sensors are generally most suited as disposable sensors.^45^ An ultra-low-cost approach for the development of robust, disposable potentiometric sensors has also been demonstrated using conventional filter papers, which were made conductive with the use of a CNT ink.^161^ In this way, CNTs acted as both the electric conductors and ion-to-electron transducers of the potentiometric signal. In comparison to metal coated papers, the conductive CNT-coated papers demonstrated a good ability to withstand mechanical stress. No significant changes in electrical resistance or mechanical properties were observed after the papers had been subjected to several physical bending and folding cycles. Another example includes a flexible and disposable paper-based pH sensor fabricated using a pencil-drawn working electrode.^162^ Despite the attractive features of paper, the infiltration of liquid can cause the substrate to bend and expand. To avoid such drawbacks, wax can be impregnated into the paper to prevent the substrate from absorbing liquid and changing its shape during pH measurement.^163^ Alternatively, a robust low-cost, flexible and disposable potentiometric ion-sensing system has been developed using Newspaper.^134^ To prevent water penetration and potential chemical leaching the mechanical and chemical stability of the newspaper based pH sensor was assured by directly coating Parylene C (PC) onto the substrate *via* chemical vapor deposition (CVD). The PC coating rendered the hydrophilic surface of the pristine newspaper hydrophobic, completely preventing chemical leaching and blocking water penetration (Fig. 2h) and yielded a network of roughened cellulose fibres but smoother pores. The PC coated papers exhibited a strong chemical resistance to both acids and bases. The coating also efficiently increased the Young's modulus and the tensile stress of the PC-papers, which were maintained even after wetting.

In addition to this, commercially available sheets of temporary transfer tattoo paper have also been used as body-compliant substrates that offer skin like elasticity.^137,150,164–166^ With screen-printing technologies, such substrates can offer an attractive platform for the design of high-performance wearable sensors.^135^ The versatility of the transfer tattoo paper facilitated an attractive design with solid-contact, and ion-selective electrodes for a non-invasive, potentiometric, epidermal pH monitoring device (Fig. 2i).^135^ Furthermore, the flexible and elastic nature of the tattoo substrate could facilitate firm attachment of the potentiometric pH sensor to almost any exposed skin area on the body.^135^ Moreover, repeated bending and stretching of the tattoo-based sensors exhibited minimal effect on mechanical integrity and potentiometric behaviour. Neither bending (180°) nor stretching (10%) strains impacted the response of the tattoo-based sensor. In addition, the sensors exhibited no apparent memory effect and could rapidly measure dynamic pH changes.

## 4. Materials and electrodes

The potentiometric electrochemical biosensors provide a simple and attractive approach for the analysis of biological samples through direct measurement of electrochemical reactions. A standard potentiometric sensor consists of a combination of two electrodes, a sensitive (working) electrode and a reference electrode (RE).^152,167–170^ To determine pH, potentiometric sensors measure the potential difference between two electrodes immersed in a solution of unknown pH (according to Nernstian equation). In this section, we discus different materials which are used for the fabrication of sensitive and reference electrodes for wearable potentiometric pH sensors.

### 4.1. Flexible reference electrodes

**Fig. 3:**
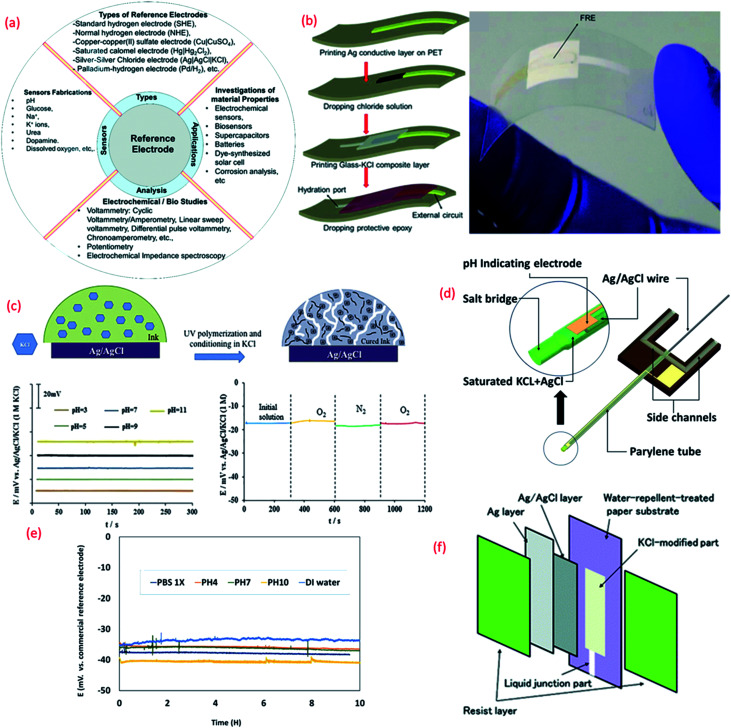
(a) Importance of reference electrode (RE).^180^ (b) 3D schematic of the fabrication of lead-free glass–KCl composite based RE layer printed on the top of an Ag/AgCl film and mechanical bending of flexible RE (Reprinted from Publication with permission from).^180^ (c) The mechanism of formation of KCl porous network in membrane structure on the top of Ag/AgCl and bottom shows the pH response of the RE in different buffer solutions and effect of oxygen presence on the RE potentiometric response Reprinted from Publication^171^ with permission from Elsevier. (d) 3D schematic of the new reference electrode with electrolyte reservoir Reprinted from Publication^181^ with permission from Elsevier. (e) Potential difference between micro flexible RE and commercial RE in different solutions, Reprinted from Publication^181^ with permission from Elsevier. (f) Schematic diagram showing the structure of the synthesized Ag/AgCl reference electrode on paper Reprinted from Publication with permission from ref. 182.

Thin or thick film Res, especially Ag/AgCl/KCl is a major component of potentiometric pH sensors (Fig. 3a). Due to the challenges related to the formation of the KCl layer, which is otherwise essential for the stability and repeatability of measurements in potentiometric pH sensors, the majority of wearable solid state thick/thin REs are quasi REs (*i.e.*, absence of KCl layer).^171,172^ Miniaturized thick film REs have been successfully fabricated using polymer Ag/AgCl, glassy AgCl, glassy Ag/AgCl, glass–KCl layers, *etc.*^173–175^ However, the lack of flexibility and the instability of some of these RE designs is a major issue, particularly in context with wearable systems. Even though they can be developed with simple fabrication steps, a majority of them exhibit fluctuations in potential while reacting with Cl^−^ ions and instability during long term operation.^176,177^ Some of the approaches reported to stabilize the potential of REs include a lead-free glass–KCl composite layer printed on the top of an Ag/AgCl film.^167,178,179^ The decay of the glass–KCl composite electrode in solution is very low and hence the fabricated thick film REs can show lifetime of over 2 years.^179,180^ The flexible REs developed with this composite material can also exhibit excellent performance (Fig. 3b),^180^ with negligible variation in potential (±4 mV) under different bending states (radius 3, 5, and 7 mm).^180^

For the large-scale fabrication of flexible REs, novel methods such as solid junction membrane based on a polyacrylate inkjet ink could be explored. In this method, inkjet printed Ag electrodes on flexible PET substrates are chemically transformed into Ag/AgCl electrodes and then the membrane cocktail is drop-casted (Fig. 3c). These electrodes show stable response in pH 3–11 (Fig. 3c) along with negligible interference oxygen present in water (Fig. 3c). Due to low processing temperature, the use of UV curable ink with KCl layer is attractive for the fabrication of stretchable or ultra-flexible miniaturized REs.^171^

**Fig. 4:**
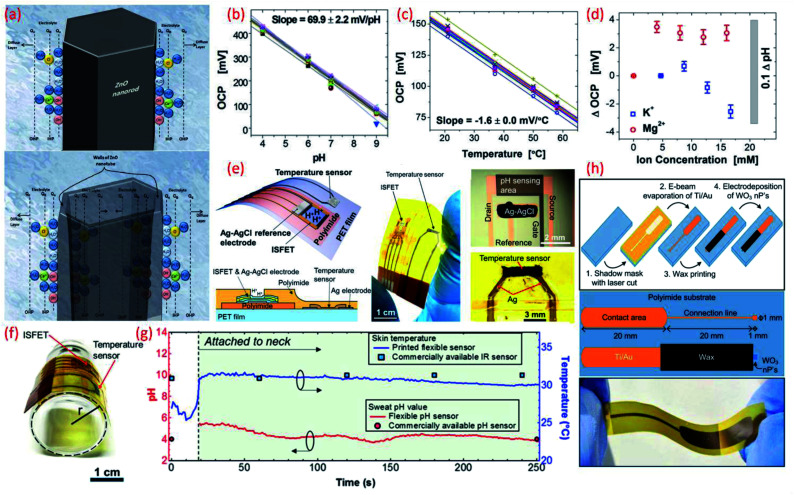
(a) Schematic diagram showing the charge distribution at the ZnO nanorods and on ZnO nanotubes–electrolyte interface.^183^ (b) Potential as a function of pH. Reprinted with permission from John Wiley and Sons.^184^ (c) Temperature dependence of potential. Reprinted with permission from John Wiley and Sons.^184^ (d) Influence of ion interference on potential measured from an array of 30 pH IrO_2_ sensing electrodes. Reprinted with permission from John Wiley and Sons.^184^ (e) Schematic of a wearable pH and temperature sensors with cross-sectional diagram of the device, photograph of the fabricated device and micrograph of the ISFET component and magnified picture showing the temperature sensor, Reprinted (adapted) with permission from^185^ Copyright (2017) American Chemical Society. (f) Image shows the flexible ISFET and temperature sensor, Reprinted (adapted) with permission from^185^ Copyright (2017) American Chemical Society (g) real-time pH and skin temperature acquired by the integrated sensors (red and blue dots represent the control experiment data for pH and skin temperature, respectively, measured using commercially available pH and IR sensors), Reprinted (adapted) with permission from^185^ Copyright (2017) American Chemical Society. (h) Scheme of the sensor fabrication, design and structure, and the photograph of the final fabricated WO_3_ based flexible pH sensor Reprinted (adapted) with permission from^186^ Copyright (2014) American Chemical Society.

Despite the challenging miniaturization of REs micro-fluidic based pH sensors have also been reported in literature. Some approaches for the efficient design of microfluidic potentiometric pH sensors include the fabrication of flexible micro-REs with an internal KCl electrolyte reservoir in the form of a parylene tube and an Ag/AgCl metal wire (Fig. 3d).^181^ In comparison to a printed film, the Ag/AgCl wire provides a larger amount of Ag and AgCl and thus extended lifetime of the reference material (Fig. 4d).^181^ Furthermore, the KCl electrolyte reservoir improves the potential stability by supporting a stable reference voltage for solutions of different pH (Fig. 3e).^181^ The integration of micro-REs with yarn or fibre-based pH sensitive electrodes^132^ could lead to microfluidic based pH sensors for sweat monitoring. Similar works that have demonstrated fluid transpiration driven by capillary forces due to the use of porous paper based substrates have been discussed in Section 3.^182^ The methods of flexible RE fabrication discussed within this section (Fig. 3f) are also useful for the efficient design of such paper-based sensors.

### 4.2. Flexible sensitive electrodes

#### 4.2.1. Metal oxide (MOx) based flexible pH sensors

**Fig. 5:**
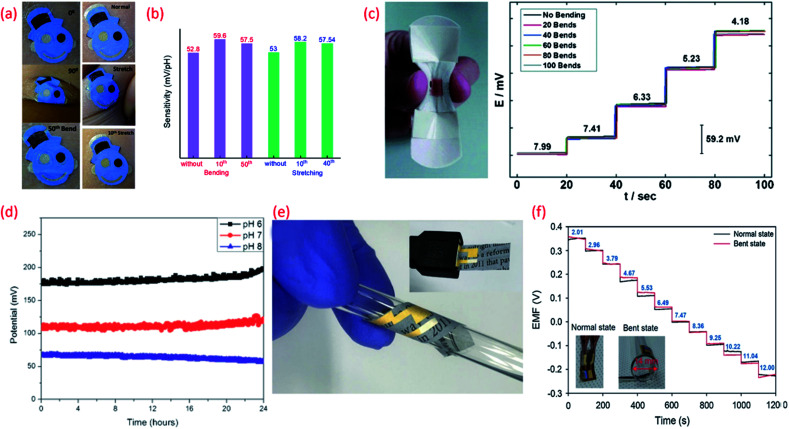
(a) Images of the tattoo pH sensor with PANi sensitive electrode applied to cubital fossa at 0° bending, 90° bending, and after the 50th bending and Images at normal, during stretching, and after the 10^th^ stretch Reprinted with permission from Publication.^210^ (b) The comparison of sensitivity variation of the tattoo-based sensors under different bending stretching condition Reprinted with permission from Publication.^210^ (c) Image of bandage-based pH sensor with PANi electrode in bending condition and its calibration curves from pH 7.99 to 4.18 (each trace taken after 20 bends) Reprinted with permission from John Wiley and Sons.^152^ (d) Long-term potential stability of the wound monitoring pH sensors (based on PANI electrode) at pH 6, pH 7 and pH 8 (pH values observed during chronic wound healing) Reprinted from Publication^206^ with permission from Elsevier. (e) Image of USB-type sensing platforms and flexible state of ion-selective sensor Reprinted from Publication^134^ with permission from Elsevier. (f) Response of pH sensors (PANI electrode) with increasing pH levels under mechanically normal and bent states (image shown in inset) Reprinted from Publication^134^ with permission from Elsevier.

MO_*x*_ have recently gained increasing interest for electrochemical and biosensing applications due to their unique electrical, electrochemical and highly sensitive properties which assert their utility in healthcare and water and food quality monitoring applications.^32,187–189^ Due to high surface to volume ratio, the morphology of nano structured MO_*x*_ enhances the sensitivity, response time, selectivity and catalytic activity of sensitive electrodes.^47^ For example, in comparison to ZnO nanorods (sensitivity 28.4 mV pH^−1^) ZnO nanotubes show high sensitivity (45.9 mV pH^−1^) due to their low dimensionality and higher levels of surface and subsurface oxygen vacancies.^183^ In addition the large effective surface area of the nanotubes provides a substantial platform for ionic interaction as shown in Fig. 4a.^183^ The difference in sensitivity is due to the surface charge distribution at two walls of the MO_*x*_–electrolyte interface.^183,190^ In our previous work we observed that the CuO nanorectangle based pH sensors have better sensitivity and stability in respect to CuO nanoflower based sensors due to high crystallinity, surface area and low surface roughness of the nanorectangular structure.^22^ Besides, the long lifetime of MO_*x*_ in different environmental conditions makes these materials excellent candidates for wearable potentiometric pH sensors. Among various MO_*x*_ used for the fabrication of pH sensors, RuO_2_ and IrO_2_ allow pH measurements over wide ranges, with fast responses, high accuracy and high durability.^32^ However, for flexible and wearable pH sensors, the application of these oxides, especially RuO_2_, is limited due to the lack of biocompatibility, flexibility, high cost and high temperature processing required.^32,188,191^ The sensitivity of MO_*x*_ based pH sensors largely depends on the material composition and the method used for material deposition because the microstructure, porosity, surface homogeneity and crystalline structure of the material influences the sensing performance (Table 3).^32,191,192^ It has been demonstrated that IrO_2_ has very good biocompatibility and proved its cell viability close to 100% which is higher than indium tin oxide (ITO).^193,194^ In addition to excellent sensitivity (the majority of reported works show super-Nernstian response due to involvement of more than one proton per electron in the electrochemical reaction^195^), the biocompatible characteristics of IrO_2_ lead to *in vivo* and *in vitro* applications. Detailed studies in this regard have been described in previous review articles.^172,200,201^ For example, pH sensitive IrO_2_ electrodes for sweat monitoring displayed a super-Nernstian sensitivity of 61 ± 1 mV pH^−1^.^195^ IrO_2_ based pH sensors have also been developed on conductive textiles by electrodeposition.^194^ Fabricated on a stainless-steel mesh, the sensors showed a sensitivity of 47 mV pH^−1^ (pH range 4–8) with a relative error of 4% as compared to commercial pH strips. The major advantage of textile-based sensors is their wearability and comfort for the wearer.^133^ However, wearable cloth-based pH sensors require further studies in terms of bacterial growth and washability. Changes in temperature and the influence of non-target ions can also cause shifts in potential between the sensing electrode and the RE. For example, an IrO_2_ based pH sensing array that demonstrated a sensitivity of 69.9 ± 2.2 mV pH^−1^ (Nernstian response for array of sensors shown in Fig. 4b) revealed a temperature influence (20–60 °C) on open circuit potential (OCP).^184^ The sensor shows a linear dependence of −1.63 ± 0.02 mV/°C (Fig. 4c) and the variation of <3.5 mV OCP for various ions (Fig. 4d). Hence, the pH sensor shows a shift in OCP ≈0.1 pH for a temperature change of 5 °C and ≈0.05 pH for ions. This study suggests that a proper calibration algorithm and a number of additional sensors can overcome issues related to the influence of other parameters^184^ The recent investigation in MO_*x*_ based pH sensors for biomedical and clinical applications is concentrated towards ISFET, extended-gate field effect transistor (EGFET)^199^ based pH sensors, both of which work on the principle of potentiometry. The major advantages of these MO_*x*_ sensitive electrodes, which include ZnO,^197^ Indium Zinc Oxide (IZO), indium tin oxide (ITO),^199^ and InGaZnO films, are their flexibility and miniaturization. For example, with InGaZnO as sensitive electrodes, the fully flexible ISFET pH sensor^185^ (Fig. 4e and f) exhibits a sensitivity of 51.2 mV pH^−1^.^185^ As per the Nernst equation, the pH potential is proportional to temperature. As mentioned previously within this section the temperature and environment of the testing solution can influence pH measurements. In terms of wearable potentiometric applications, skin temperature could affect pH measurements but this could be overcome by integrating an ISFET and temperature sensor on the same substrate, as shown in Fig. 5e.^185^ The real time experiment with these sensors attached to the user's neck reveal that both the pH and temperature sensors exhibit excellent performance in comparison to commercial sensors with a small fluctuation in pH level (Fig. 4g(ref. 185)). In comparison to potentiometric sensors, the use of ISFETs is limited by their high operating voltage. Amorphous oxide based transistors^197^ have also been used as sensors due to their excellent electrical properties, low processing temperature, high reliability and easy reproducibility.^198,202^ For example, flexible electrolyte-gated neuron transistors with amorphous oxide (IZO) channel layers have been reported for biochemical pH sensing applications.^198^ With strong electrical double layer (edl) modulation at the electrolyte/oxide interface, the electrolyte gated transistors are able to operate at low voltage and thus are more suitable for portable systems.^198^ Such IZO based neuromorphic transistors show that the dual gate synergic modulation mode could offer improved sensitivity (105 mV pH^−1^*versus* 37.5 mV pH^−1^ with single gate mode).^198^

**Table 3:** Properties of metal oxides based pH sensors for wearable/flexible electrochemical sensor fabrication

Material	Fabrication	Substrate	pH range	Response time	Sensitivity (mV pH^−1^)	Flexibility	Ref.
IrO_2_	Sputtering	PET	4–7	—	61 ± 1	—	195
IrO_2_	Electrodeposition	PI	4–9	0.5 s	69.9 ± 2.2	—	184
IrO_2_	Sol–gel	PI	1.5–12	0.9 to 2 s	51	Tested in a tube with a 1 cm curvature radius	196
CuO nanorectangle	Hydrothermal synthesis	PET	5–8.5	—	0.64 μF pH^−1^	Tested in a tube with a 5 mm curvature radius	22
ZnO nanowalls	Low temperature	PI	1–9	—	∼59	—	197
	Polycrystalline silicon thin film transistor technology						
IZO	Sputtering	PET	4–10	5 ms	105	Tested by bending around a cylinder with a 1.0 cm curvature radius	198
ITO	Radio frequency sputtering and a roll-to-roll process	PET	2–12	—	50.1	—	199
InGaZnO	Sputtering and thin film transistor technology	PI	3.3–11	—	51.2	Tested as a function of bending with up to 13 mm curvature radius	185
WO_3_ nanoparticle	Electrodeposition	PI	5–9	23–28 s	−56.7 ± 1.3	—	186

High surface area materials have also been investigated for cost-effective, lightweight flexible potentiometric pH sensors. For example, WO_3_ nanoparticles electrodeposited on a polyamide substrate have been used for the development of a wearable pH sensor for biomedical applications.^186^ These flexible electrodes have been shown to exhibit a sensitivity of 56.7 mV pH^−1^ in the range of pH value 9–5 (Fig. 4h) with sensor response time of 23–28 s in the pH range 9–5.^186^ A summary of the sensing performances of MO_*x*_ based pH sensors is given in comparison Table 3. Lack of ultra-flexibility, high temperature processing and high cost are some of the limitations of MO_*x*_ based flexible pH sensors. Such issues have been addressed with the use of polymer or carbon as the sensitive materials, as described in the following sections.

#### 4.2.2. Polymer based flexible pH sensors

Polymer based organic conductors show very good pH sensing performance due to their excellent electrochemical properties including the variation in oxidation states and ion-exchange (Table 4).^203–205^ For example, polyaniline (PANi) conducting polymer has a variety of oxidation states which are pH and potential dependent. The reversible transformation of emeraldine salt (ES) and emeraldine base (EB) during acid–base reaction is associated with the pH sensitivity of PANi.^205^ In acidic solutions, the polymer is doped with H^+^ ions to create the electrically conductive ES form of PANi and the resulting surface charge increases the electrical potential between the sensitive and reference electrodes. In the case of alkaline solutions, captured H^+^ ions are neutralized or deprotonated, leading to the formation of the EB form of PANi which displays a decreased polymer surface charge/potential due to the non-conductive nature of this PANi phase.^206^ The pH sensitivity of the PANi electrode strongly depends on the polymerization condition of the polymer^206^ however, PANi generally exhibits super Nernstian response^207,208^ and fast response time.^209^ Even though the polymers show limited chemical stability and low mechanical strength their high flexibility, stretchability and easy deposition on any flexible substrate is attractive for wearable sensors. In addition, deformation of the PANi film as a result of the stretching/bending (Fig. 5a) could be advantageous and may even improve the sensing performance of the electrode.^210^ A comparison (drawn from the data presented in the reported work) of the influence of stretching on the sensitivity of PANi (Fig. 5b) shows that enhanced sensitivity during stretching originates from increased conductivity of the film due to uncoiling and reorientation of the crystalline and amorphous phases of PANi.^210^ The response of the PANi sensitive electrode is also influenced by the thickness of the film. The thinner the PANi layer the faster the response time, in comparison to thicker films deposited by drop casting methods.^206^

**Table 4:** Properties of polymer and carbon-based pH sensors for wearable/flexible electrochemical sensor fabrication

Material	Fabrication	Substrate	pH range	Response time	Sensitivity (mV pH^−1^)	Flexibility	Ref.
PANi	Electrodeposition	a. PET	1–13	A few seconds	58	—	125
		b. PVC coated steel wire					
PANi	Electrodeposition	PDMS	4–7	∼60 s	—	Tested by mechanical friction and skin deformation	131
PANi	Drop-casting	PET-coated palette paper	4–10	Rise time 12 s	50–58.2 (pH 2–12)	—	206
				Fall time 36 s (pH 6–8)			
PANi	Electropolymerization	Commercial adhesive bandage	5.5–8	20 s	58.0 ± 0.3	Tested by flexing the sensor and then releasing the device to return to its unperturbed state (100 iterations)	152
PANi	Electropolymerization	Commercially available temporary transfer tattoo paper	3–7	25 s	52.8–59.6 (dependent on bending/stretching conditions)	Tested using GORE-TEX under 50 bending (180°) and 40 stretching (10% in lateral extent) applications	210
PANi	Electrodeposition	Parylene C-coated newspaper	2–12	<10 s	58.2	Tested on a glass rod with respect to a bending radius of 7 mm	134
PANi	Dilute chemical polymerization	PET	3.9–10.1	12.8 s	62.4		7 and 126
PAA-CNTs	Electropolymerization	Si-chips	2–12	3 s	54.5	—	212
SWCNT	Vacuum filtration method	PET	3–11	30 s	59.71	Tested by measuring resistivity upon hard bending	8 and 211
G-PU	Printing	PDMS	5–9	8 s	11.13 ± 5.8	Tested by measuring resistance under 500 stretching cycles (30% strain), with the use of a stepper motor	8
G-PU	Printing	Cellulose-polyester blend cloth	6–9	5 s	47 ± 2	Demonstrated by hard crumpling	133
						Tested under 500 bending cycles at 11.40 mm bending radius	

The biocompatibility and flexibility of PANi are also attractive features of the pH sensitive material for wearable healthcare applications.^152^^,^^206^ As previously discussed in Section 3 PANi based pH bandage sensors (Fig. 5c) have been shown to have a sensitivity of 58.0 ± 0.3 mV pH^−1^ in the pH range 4.35 to 8, little interference from other ions (Na^+^, K^+^, Cl^−^, SO_4_^2−^), good reproducibility and lack of hysteresis effect. Such bandages can detect the variation of pH at a wound site for up to 100 minutes. However, chronic wounds can take anywhere between a few days to weeks to heal and in certain cases can become long-life ailments. In such cases, the operational lifetime and mechanical stability of the PANi needs to be improved. In this regard, chemically modified PANi pH sensitive electrodes could offer good operational stability and long shelf life.^208,209^ For wound monitoring and assessment, the drift in potential of PANi pH sensing electrodes is also a concern. For example, it is found that a drift in the output voltage of 0.5 mV h^−1^ or 0.01 pH h^−1^ (due to leakage of the electrolyte from the polymer matrix covering the Ag/AgCl RE) occurs (Fig. 5d) after the first five hours of measurement.^206^ Considering the need to replace a wound bandage/dressing material every 24 h, such PANi based sensors hold significant promise for a range of wound related applications. PANi sensitive electrodes have also been used for the development of ultra-flexible and biodegradable pH sensors on paper substrate^134^ (Fig. 5eE). The low-cost, disposable, and mechanically and chemically stable ultra-flexible pH sensors demonstrated Nernstian sensitivity of 58.2 mV pH^−1^ (normal and bend state) (Fig. 5f). The sensors also exhibited good ion selectivity with a preference for H^+^ in the presence of K^+^, Na^+^, NH_4_^+^, Ca_2_^+^, and Mg_2_^+^ interfering cations, and a response time of <10 s to reach 90% of the equilibrium potential value. In addition, the sensors presented a low potential drift rate of 1.4 mV h^−1^.^134^

#### 4.2.3. Carbon based flexible pH sensors

**Fig. 6:**
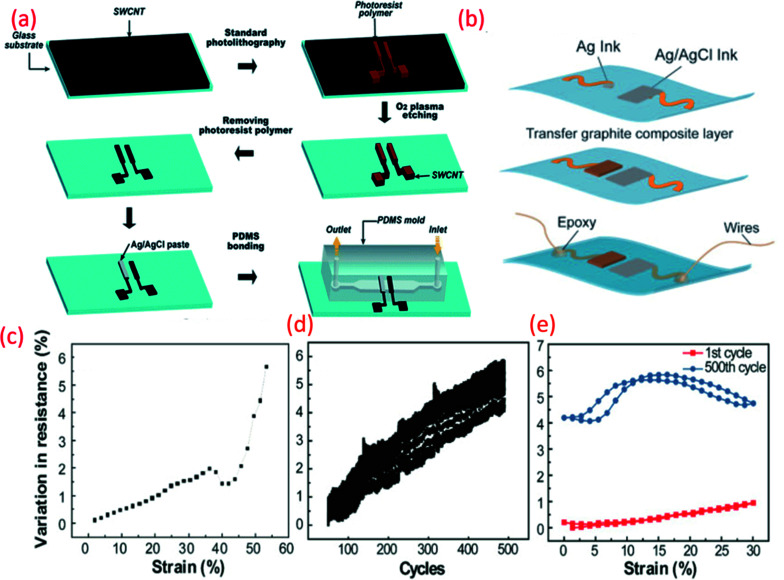
(a) Schematic fabrication process of the microfluidic pH-sensing chip with SWCNT as sensitive electrode Reprinted with permission from Publication.^211^ (b) Schematic diagram of paper electrode structure for carbon-based pH sensor (ChrPr: chromatography paper, RE: reference electrode, PCE: pencil carbon electrode).^162^ (c) Potential variation with pH for the carbon-based sensor (calibration curve).^162^ (d) G-PU based sensitive electrode on stretchable substrate for sweat monitoring Reprinted from Publication^8^ with permission from Elsevier (e) the variation in resistance across pH sensing G-PU electrode and stretchable interconnect with respect to the external strain, cyclic stretching test (up to 30% strain) and comparison in resistance variation between the first cycle of stretching and releasing and 500^th^ cycle, Reprinted from Publication^8^ with permission from Elsevier.

Carbon-based electrodes have good potential for disposable and low-cost wearable pH sensors. The attractive electrochemical features of single- and multi-wall carbon nanotubes (SWCNTs and MWCNTs) including potentiometric stability and good electrical, thermal, chemical and mechanical properties hold promise for flexible potentiometric pH sensors.^211^ Taking advantage of these features, microfluidic pH sensing chips based on SWCNTs deposited on both glass and flexible (PET) substrates have been developed^211^ with an Ag/AgCl RE painted on one of the SWCNT electrodes (Fig. 6a). The fabricated electrodes exhibited a sensitivity of 59.71 mV pH^−1^ between pH 3 and 11 with a standard deviation of sensitivity of 1.5 mV pH^−1^. Such sensors are suitable for flow analysis measurement and the detection of metabolic processes in biological cells.^211^

Carbon-based electrodes have good potential for disposable and low-cost wearable sensors. For example, simple solutions such as flexible and disposable paper-based pH sensors with pencil-drawn working (sensing) electrodes and an Ag/AgCl paste RE have been reported.^162^ The pH sensitivities of this paper-based pH sensor ranged from 16.5–26.9 mV pH^−1^.^162^ One of the issues with such sensors is that the quasi RE may have strong influence in the Cl^−^ ions in the solution. This could be overcome by using KCl layer on the top of the Ag/AgCl layer (discussed in Section 3.3).

#### 4.2.4. Polymer-carbon blend flexible pH sensors

Even though polymer-based pH sensors show excellent sensitivity, one of the major problems with these sensing systems is their long-term chemical and mechanical stability. In this regard, the combination of carbon nanotubes (CNTs) and polymeric materials such as poly(1-aminoanthracene) (PAA) have shown some promising results by stabilizing the polymer's response and increasing the lifetime of the pH sensor to over 120 days.^212^ Additionally, it has been revealed that PANi coated CNTs exhibit a significant improvement in performance, particularly in terms of linear response time and reproducibility in comparison to pure CNT based pH sensors.^125^ The PANi/CNT pH sensor also demonstrated a lower drift and hysteresis effect and was also highly selective to H^+^ ions.^125^

As we have briefly mentioned in Section 4 our previous work, focused on the development of stretchable G-PU potentiometric pH sensing electrodes and an Ag/AgCl paste RE (Fig. 6b).^8^ The stretchable pH sensor demonstrated a sensitivity of 11.13 ± 5.8 mV pH^−1^ with a maximum response time of 8 s. Furthermore, interfering ions and analytes, including Na^+^, K^+^ and glucose had a negligible effect on the performance of the pH sensor. The stretchable wireless system itself could withstand 30% strain, the average strain experienced by human skin, for more than 500 cycles (Fig. 6c). The excellent ionic conductivity of PU also assists the electrochemical reaction of the electrode.^213^ When the G-PU composite is exposed to an acidic or alkali solution, an EDL is formed at the electrode-solution interface. Consequent changes in the sensor's electrical properties, such as impedance and capacitance alter the surface potential of the electrode which, in addition to pH variation, heavily influences the electrochemical measurements.^40,214^ As well as this, the soft domain units and urethane group oxygen atoms of PU enhance the electrode's electroactive surface area.

## 5. Sensor designs suitable for other applications

**Fig. 7:**
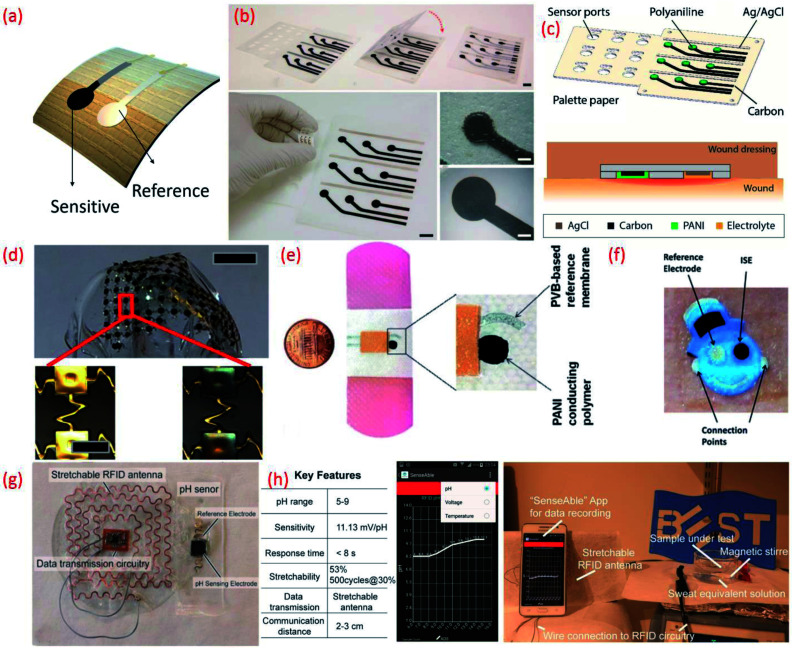
(a) Image of flexible potentiometric pH sensor (b) photographs of the fabricated wound monitoring pH sensor array on paper substrates with dimension and size of the electrodes Reprinted from Publication^206^ with permission from Elsevier. (c) A 3D schematic view of the 3 × 3 wound monitoring pH sensor arrays on paper with self-aligned encapsulation and its cross section showing the sensor embedded into a wound dressing Reprinted from Publication^206^ with permission from Elsevier. (d) Picture of an array of pH sensors with magnified images in the lower panels show gold electrodes before (left) and after (right) electroplating IrO_*x*_ Reprinted with permission from John Wiley and Sons.^184^ (e) Images of the printed potentiometric sensor on an adhesive bandage Reprinted with permission from John Wiley and Sons.^152^ (f) Image of Smiley Face tattoo pH sensor Reprinted with permission from Publication.^135^ (g) Stretchable RFID antenna and pH sensors on PDMS substrate, Reprinted from Publication^8^ with permission from Elsevier. (h) Screenshot of smartphone App “SenseAble” and photo of real-time pH monitoring system including stretchable pH sensor in sweat equivalent solution, stretchable antenna and mobile monitoring App, Reprinted from Publication^8^ with permission from Elsevier.

Depending on the application, in wearables the standard design of potentiometric sensors (Fig. 7a) could have different shapes or use an array of electrodes.^152,167–170^ Considering the requirements of wearble systems, integrated sensing technologies should be ultra flexible and/or stretchable. In addition, wearable devices for clinical diagnostics, such as those used for remote or real-time healthcare monitoring require the following features: (i) reduced measurement errors and testing time (ii) lack of interference from other analytes (iii) high performance under bending conditions (iv) adequate availability of bio fluids (*e.g.* sweat, tears *etc.*), (v) full system biocompatibility, *etc.* Considering these requirements, potentiometric sensors with various shapes and designs are needed (depending on applications including sweat, wound monitoring *etc.*) and few of them have been reported for wearable applications.^8,196,210,215^

A sensing array using multiple individual sensors can be advantageous in terms of reduction in measurement errors and testing time, and could also facilitate the mapping of multiple analytes. For example, distinct responses to buffer solutions of different pH and the time points for transition events was clearly demonstrated using 16 individual IrO_*x*_ based sensing electrodes (Ag/AgCl RE) on a single polyamide flexible substrate.^216^ In addition to healthcare applications, potentiometric pH sensing arrays also find application in real time water quality monitoring. For instance, more than 6 months of continuous water monitoring has been demonstrated using an array of RuO_2_ based pH sensing electrodes (sensitivity 55.64 mV pH^−1^).^179,217^ However, the lack of flexibility and cost of the electrode are some of the bottlenecks associated with this sensor design. The cost of the sensing array may be overcome by combining RuO_2_ with inert oxide based binary electrodes.^37,167,179,218^ For example, a low-cost potentiometric sensing array fabricated by screen printing a composite ink consisting of graphene platelets and submicron RuO_2_ powders has been reported.^219^ This approach also addresses the issues related to flexibility.^219^ In this pH sensing array, additional ions that can be found in sweat, including Ca^2+^, Na^+^, K^+^, *etc.* could also influence the sensing performance. This issue can be evaded by applying a Nafion polymer membrane coating on top of the sensitive electrode. The addition of this membrane can prevent redox-interference and improve the durability of the MO_*x*_ by preventing delamination or dissolution of the sensitive electrode.^220–222^ Data fusion and fault diagnosis can also help to improve the accuracy and reliability of the flexible potentiometric pH sensing arrays based on RuO_2_ sensitive electrodes and Ag differential REs.^223^

Flexible sensing arrays can also facilitate spatial mapping of wound pH, which can reveal the location and severity of bacterial infections. For example, the simultaneous measurement of multiple sites within one wound region was made possible with an inexpensive flexible array (3 cm × 3 cm) of pH sensors on a palette paper substrate (Fig. 7b and c).^206^ The wound monitoring device, fabricated using an Ag/AgCl RE and a sensitive electrode based on carbon coated with a conductive polymer, PANi could continuously monitor the pH levels of the wound site for 5 h.^206^ Spatial endocardial pH distribution mapping of a human heart under-going ischemia has also been demonstrated using flexible pH sensing arrays (Fig. 7d).^184^ This study also noted that contact uniformity between the sensor and the heart's surface is important and should be considered in future device designs. The pH-sensitive bandages that directly attach to the human body for wound monitoring (Fig. 2e)^152^ is one of the best examples of the efficient design and application of wearable potentiometric pH sensors using polymeric materials. The minimal influence of bending (100 iteration) and twisting on the performance of sensors embedded in the pH-sensitive bandage suggests that they are resistant to mechanical stress.^152^

The sensor designs are sometimes fashioned into anaesthetically pleasing “Smiley Face” with solid-contact, ion-selective electrodes to shape a non-invasive, potentiometric, epidermal pH monitoring device (Fig. 7f). For example, the indicator and RE eyes of the “Smiley face” could be printed using carbon and Ag/AgCl inks, respectively. The bare electrodes were further modified using pH responsive PANi. The ears of the smiley face could contain the contact pads for electronic readout. A KCl-saturated insulative layer could isolate the underlying Ag/AgCl inter-connects between the electrodes (eyes) and contact pads (ears). The resulting potentiometric sensor exhibited a rapid and sensitive response to a wide range of pH changes, demonstrating a sub-Nernstian response (50.1 mV pH^−1^) in the physiologically relevant pH range of 3–7 and stable signals even when operating under profuse perspiration.^135^ For wearable applications, the pH sensor requires durable and compatible substrates that demonstrate a degree of flexibility and stretchability.^101,102,224^ Such features also facilitate the conformal integration of sensing systems into wearable devices that are designed for use with non-planar surfaces. We developed a new approach for the design of stretchable using a pair of serpentine-shaped stretchable interconnects. In this new design a wireless system is achieved by the integration of a flexible electronics circuit and stretchable (20% strain) radio-frequency-identification (RFID) antenna (Fig. 7g). The pH data was wirelessly and continuously transmitted to a smartphone through a custom smartphone App, “SenseAble” (Fig. 7h).^8^ In addition to wearable technologies, flexible potentiometric pH sensors are also relevant for *in vivo* medical diagnostic applications. The flexible IrO_*x*_ based pH sensor^196^ with radius of curvature of 1 cm has great application in small confined tunnel, for example *in vivo* reflux detection in human oesophagus. For monitoring gastroesophageal reflux diseases it is necessary to monitor pH values for an extended period in both the stomach and esophagus.^225,226^ Based on this a battery-less and wireless, implantable pH sensor integrated with an impedance sensor for gastroesophageal reflux monitoring has been reported.^225^ In this work, the impedance sensor was used to accurately detect the occurrence of reflux episodes of both acidic and non-acidic media.^225^ For the practical application of the sensor, live pigs under anaesthesia were used. The fabricated miniaturized transponder does not require a battery and is small and compliant enough to be implanted on the wall of the oesophagus using an endoscope.^225^

The continuous monitoring of blood and brain tissue pH levels are also important for patients who have suffered a stroke and/or traumatic brain injury. In this regards, fibre optic and electrochemical pH sensors have been explored with thin multilayer coatings of titanium and iridium oxide on flexible Kapton substrates.^227^ The electrochemical pH sensor (Nernstian response of 57.9 ± 0.3 mV pH^−1^ in the range of pH value 6.8–8 with response time less than 5 s) can be inserted into the skull of patients of traumatic head injury. For *in vivo* analysis, the sensing performance was examined in Sprague-Dawley rats, with the sensor showing comparatively negligible drift.^227^ In addition to sensor designed aimed at reducing errors, measurement time and improving mapping *etc.*, new pH sensor designs must also overcome issues relating to lack of electrolytes/sweat due to the poor collection of body fluids. In Section (6.2), we discuss the potential methods that have been developed to defeat such issues.

## 6. Challenges of flexible potentiometric pH sensors

### 6.1. Power supply

**Fig. 8:**
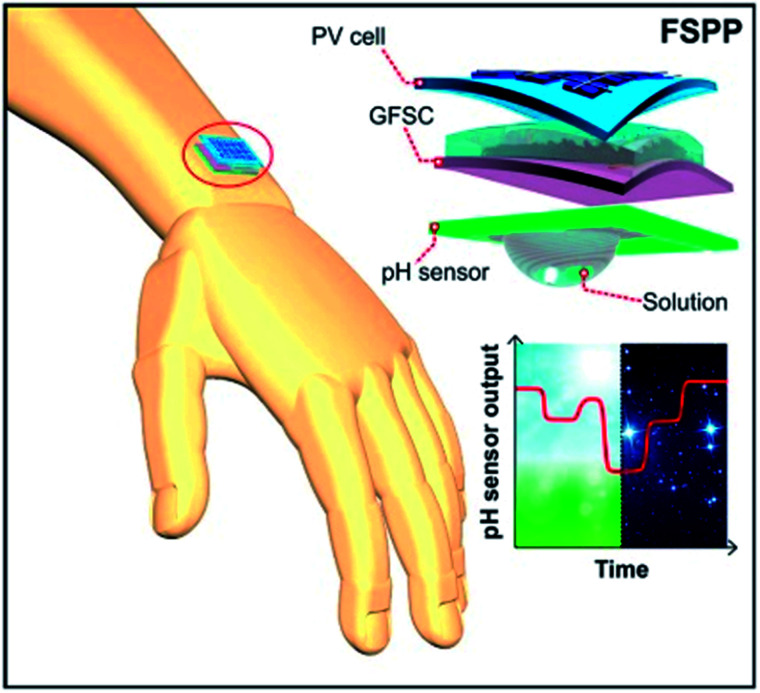
Schematic representation of fully flexible self-powered wearable pH sensor.^5^

For the continuous operation of wireless wearable systems, flexible, low powered electronics and biocompatible power sources are desired (Fig. 1). Recent advances have witnessed the integration of wearable sensing platforms with wearable energy systems for power supply.^5,228^ In our previous work, we developed a fully self-charging power pack (FSPP) for wearable applications (Fig. 8).^5^ Power generated from a flexible solar cell was stored in flexible graphene foam-based supercapacitor (GFSC). The stored power was used to operate a chemi-restive CuO based flexible pH sensor. We confirmed that the FSPP could be used for 24 h of continuous sweat pH monitoring.^5^ In comparison to chemi-resistive sensors, the potentiometric sensors do not require an operating power. Instead, the proposed FSPP can be used to power the electronics of the textile-based pH sensors in wearable sweat monitoring devices.

### 6.2. Lack of sufficient body fluids for reliable pH measurements

As we mentioned in Section 2 the pH value of skin is related to the pH of sweat. However, the major drawbacks of sweat pH sensing for clinical diagnosis or to track physiological performance during exercise includes a lack of sweat production or sweating and poor sweat collection which thus results in a poor flow of sweat to the sensing area, in addition to sweat rate, *etc.* Sweating may vary from person to person and the rate of sweating depends on the individual's health condition, physiological status and the level of exercise conducted, *etc.* Nie *et al.*^77^ introduced a flexible microfluidic device with an integrated silicon sensor chip for electrochemical pH monitoring of sweat. The miniaturized, silicon-based chips offered high accuracy and sensitivity as well as reliable and precise sensing. For flexible sensors that attach to the human skin and/or those integrated in bandages, polymer foil based flexible electronics are most suitable, including Si chips and microfluidic devices. To overcome issues with the continuous flow of sweat to the sensing area of a body compliant pH sensing chip, a cellulose paper based, flexible microfluidic device that operated using an evaporative pumping mechanism was developed. Here, the sweat is collected from the surface of the device and transported through microchannels by capillarity forces and finally directed toward the device outlet through a porous structure using evaporative pumping. This approach ensures the continuous flow of sweat to the sensing area. Bandodkar *et al.*^229^ introduced a piece of cellulose paper that acted as a perspiration sink in a tattoo-like wearable sweat sensor that contained a fluidic channel for passive sweat collection for the efficient measurement of Na^+^ ion concentration.^229^ The major advantage of the foil technology for microfluidic devices and for straightforward sensor integration, is the low production cost and the roll to roll technology enables high volume device production.^77^ In the reported work Nie *et al.*,^77^ the pH sensor fabricated using IrO_2_ (by sputtering method) as a sensitive electrode and Ag/AgCl RE. The fabricated sensor shows super-Nernstian response (61 mV pH^−1^) in the pH range 2–10.^77^ To develop precise, sensitive and miniaturized sensors in large area,^7^ a contact printing of nanowires (based on biocompatible MO_*x*_ such as ZnO) on Si chip will be advantageous, as has demonstrated this in our recent work.^230^

In the case of a lack of sweat, sweat extraction can be carried out by clinically proven techniques such as iontophoresis, a method successfully employed by Emaminejad *et al.*, in a multisensory patch designed for sweat monitoring.^215^ For easy absorption of sweat from the body during exercise, the sweat sensor can be fabricated on cellulose cloth. The high absorption rate and capillary action of the cellulose cloth will easily lead the low volume of sweat to the designated sensing area. The deposition of low temperature processable sensing materials on the top of non-conductive cloth can be achieved by screen printing carbon or polymer based sensitive electrodes.^133^ In addition to the lack of sweating, lack of proper contact between the sensitive electrode and the top layer of the skin's surface may influence measurements of sweat pH. This problem can be overcome by using flexible, hydrogel embedded pH-sensors.^47^ Contact between the sensing interface and the skin was achieved in a non-invasive way using a hydrogel film. This thin film was enough to provide a short path to the electronic components of the sensor. The high sensitivity of the hydrogel film embedded sensor was achieved using metalized electrospun fibres of palladium/palladium oxide which have high sensitivity and electrocatalytic properties.^47^

### 6.3. Biocompatibility

Wearable technologies offer unprecedented opportunities to tackle pressing societal challenges. Diverse body compliant devices can provide solutions in the areas of point of care diagnostics, remote health monitoring, safety at work, emergency management, productivity enhancement, home energy management and others. However, the safety and security of these devices is vital.^231^ Considering wearable sensing platforms are intended for direct exposure to the user's skin and body, for extended periods of time these devices are expected to be safe. They should also possess the capacity to conform to the user's daily activities and should not pose additional safety risks or lifestyle restrictions.^232–235^

The rapid advancement of flexible electronics and the recent use of natural biomaterials, such as sodium alginate, silk, chitin, cellulose, *etc.*, as the active components and substrates of flexible sensing systems has accelerated the development of more user-friendly wearable devices.^236–243^ These elastic, biocompatible substrates prevent direct contact between the user and integrated sensors, evading the possibility of skin irritation.^244–246^ However, interactions between the human body and a single substance is difficult to predict due to complex biological reactions. Furthermore, wearable devices tend to include many components other than a substrate, sensitive material and electrodes, including a microcontroller, wireless communication system and a power source. The complexities of these systems could therefore harbour several unknown safety risks. For instance, many of the metal materials contained within wearable devices in the form of electrodes, network ports or those found within conductive textiles can cause allergic responses during extended periods of skin contact. The prolonged use of such devices can also cause bacterial buildup. Another consideration is the manufacturing process. Certain processing techniques may alter once biocompatible materials yielding incompatible or unsafe devices. In addition, due to high switching speeds, the electronic circuitry embedded in wearable sensors may stimulate elevated operating temperatures. Increased operating temperatures may be harmless during intermittent use but can cause burns or other kinds of tissue damage during longer periods of exposure. The risk of electrical shock due to the potential leakage of current from worn or defective circuitry should also be considered. Lastly, wearable devices may be prone to reactions such as galvanic corrosion when electrical currents are regularly exposed to body fluids such as sweat. Without proper safety measures, products that hold vast potential to improve our daily lives may also present unnecessary hazards.

On the other hand, the potential safety risks associated with any wearable device can be easily avoided through precautionary design and a subsequent safety evaluation. The safety features of devices designed to routinely encounter human tissue can be assessed using ISO 10993, a biocompatibility standard provided by the International Organization for Standardization (ISO). Despite the availability of biocompatibility testing standards and guidelines, product safety is often the last factor considered in the rapidly expanding field of consumer wearables. Surprisingly few reports evaluate the safety of wearable pH sensors and instead highlight the biocompatibility of the native materials that will likely be transformed during the device manufacturing process.

More recently, the safety of some wearable pH sensors targeted toward biomedical and health care applications have been evaluated. The biocompatibility of the previously described palette paper based pH sensor for the assessment of chronic wound beds was evaluated in the presence of human keratinocytes (HaCaT).^206^ The viability of the HaCaTs was assessed using a calcein AM/ethidium homodimer-1 live/dead® assay, whereas prolific growth was determined using PicoGreen® DNA quantification assay. Thought the HaCaTs did not adhere to the Ag, carbon, PANi or SE, all cells cultured in the presence or absence of the examined sensors exhibited a healthy morphology and >90% viability after 7 days of culture. Cellular DNA content also increased successively over the 7 days of culture, indicating the retained proliferation capacity of the HaCaTs. Furthermore, DNA concentrations did not vary between untreated cells and those exposed to each of the sensor materials.

The biocompatibility of a low cost and flexible pH sensor with a CNT-based miniaturized serpentine sensing element printed on top of Ag electrodes was evaluated using murine myoblasts (C2C12).^247^ The chemiresistive pH sensor was fabricated using an Aerosol jet printing technique for real-time pH monitoring in live cell applications. The pH sensor demonstrated a 20 s response time with good sensitivity (up to 59 kΩ pH^−1^), repeatability (coefficient of variance < 1.15%) and excellent biocompatibility. The C2C12 cells retained >95% viability and layers of cells continued to grow on the surface and edges of the sensor after 7 days of culture.

Finally, the biocompatibility of highly conductive and flexible cotton fibers coated with poly(3,4-ethylenedioxythiophene)-poly(styrene sulfonate) (PEDOT:PSS) and MWCNTs was investigated to determine its feasibility of use as a pH sensor for real-time wound and skin analysis.^149^ The solid-state wearable pH sensor achieved rapid and selective Nernstian responses (−61 ± 2 mV pH^−1^) over a wide pH range from 2–12. Furthermore, the deposition of pH sensitive PANi yielded electrodes with significant biocompatible and antibacterial properties. Initially, there was a significant decrease (*p* < 0.05) in the viability of HaCaT cells after a 24 h exposure to PEDOT-MWCNT fibers. In contrast, the PANi-PEDOT-MWCNT-cotton fibers did not induce any significant changes in cell viability. The PANi, which has previously been demonstrated as a biocompatible polymer, enveloped the PEDOT-MWCNT-cotton fibers and acted as protective casing to subdue the toxicity of pristine CNTs and maintain cellular viability.^135,149,248,249^ The continued growth and success of the wearable device industry will ultimately provide global access to advanced technologies. Nonetheless, a gap in the overall safety coverage of wearable devices remains, one that could expose users to unnecessary risks. To ensure the success of this industry a thorough understanding and evaluation of all the potential safety risks and hazards associated with all materials and substrates involved in a wearable device should be of prime focus.

## 7. Conclusions and future perspectives

Flexible or wearable potentiometric pH sensors have great importance for early stage determination of many chronic diseases. In this review article, we summarize the recent progress in flexible potentiometric pH sensors for healthcare applications. The major focus areas of this review are (i) the importance and (ii) the design and components of flexible potentiometric pH sensors (including, substrates, reference electrodes and various sensitive materials) (iii) the additional applications and challenges in addition to (v) the future outlook of flexible potentiometric pH sensors. We provide a detailed discussion on the challenges, including insufficient sources of power, lack of sufficient body fluid collection and sensor biocompatibility.

The future scope of flexible potentiometric pH sensors as wearable systems for clinical diagnosis and healthcare applications needs to be considered carefully. In human blood, pH range is very crucial with normal levels ranging from 7.35–7. If blood pH values vary below 6.8 or above 7.8, cells stop functioning. So, it is essential to fabricate an accurate pH sensor for monitoring blood pH in a very small range of pH variation (less than 0.05 unit). However, in wearable sensors the direct measurement of blood pH value is difficult. A strong study to explain the correlation between the pH of blood and sweat pH is required. However, the variation of glucose levels and other ions found in blood may also influence changes in pH values. To ensure the performance and selectivity of flexible potentiometric pH sensors miniaturized sensing arrays are required for body fluid monitoring. Considering the increasing demand to monitor very low units of the pH values of body fluids highly sensitive materials are required. RuO_2_ or IrO_2_ are excellent sensitive materials with long term stability. However, biocompatibility, flexibility and costs are of concern. Mixing these oxides with other materials, inert oxides or biocompatible conductive materials may overcome the above issues.^250–254^ Printing techniques are also an attractive approach to ensure the biocompatibility and flexibility of miniaturized potentiometric pH sensors. For this purpose contact printing of MO_*x*_ will be an excellent method due to its possibility of fabrication high performance sensors along with the possibility for the development and integration of low-power, miniaturized electronic devices over large areas on flexible substrates.^132,230^

The biocompatibility of wearable sensing technologies significantly improves device intimacy and facilitates secure integration with dermal interfaces. Accordingly, the biocompatibility of the materials and substrates used to develop potentiometric flexible pH sensors is a major concern. However, the potential safety risks associated with any wearable device are easily avoided through precautionary design and a subsequent safety evaluation. One reasonable way to improve sensor biocompatibility is to select natural biomaterials such as sodium alginate, silk, chitin, cellulose and even wood for the design of potentiometric flexible pH sensor design. In addition, the potential safety risks associated with each component of flexible potentiometric pH sensors, including all materials and substrates, should be evaluated in accordance with ISO 10993 biocompatibility standard testing regulations.

In addition, to realize an efficient and compliant wearable real time biofluid monitoring potentiometric pH sensor, it is important to solve the issues related to a lack of sweat collection and to develop flexible electronic circuits with wireless systems as shown in Fig. 1.

## Conflicts of interest

There are no conflicts to declare.

## Supplementary Material
