# Correction: Flexible potentiometric pH sensors for wearable systems

**DOI:** 10.1039/d0ra90022b

**Published:** 2020-03-30

**Authors:** Libu Manjakkal, Saoirse Dervin, Ravinder Dahiya

**Affiliations:** Bendable Electronics and Sensing Technologies (BEST) Group, School of Engineering, University of Glasgow G12 8QQ UK Ravinder.Dahiya@glasgow.ac.uk

## Abstract

Correction for ‘Flexible potentiometric pH sensors for wearable systems’ by Libu Manjakkal *et al.*, *RSC Adv.*, 2020, **10**, 8594–8617.

The authors regret that the funding information was not included in the acknowledgements in the original article. The following acknowledgements should have been included:

This work was supported in part by the Engineering and Physical Science Research Council (EPSRC) through Engineering Fellowship for Growth—PRINTSKIN (EP/M002527/1) and neuPRINTSKIN (EP/R029644/1) and EPSRC ORCA – Partnership Resource Fund Project MUSES (EP/R026173/1). In addition, this work received support from the European Union’s Horizon 2020 Research and Innovation Programme (AQUASENSE Project) under the Marie Skłodowska-Curie grant agreement No: H2020-MSCA-ITN-2018-813680.

The Royal Society of Chemistry apologises for these errors and any consequent inconvenience to authors and readers.

## Supplementary Material

